# Presence of human papillomavirus in breast cancer and its association with prognostic factors

**DOI:** 10.3332/ecancer.2015.548

**Published:** 2015-06-25

**Authors:** Andreína Fernandes, Gino Bianchi, Adriana Pesci Feltri, Marihorgen Pérez, María Correnti

**Affiliations:** 1 Laboratorio de Genética Molecular. Instituto de Oncología y Hematología, MPPS, Caracas 1050, Venezuela; 2 Sección de Patología Mamaria, Instituto de Anatomía Patológica José O’Daly, Universidad Central de Venezuela, Caracas 1050, Venezuela; 3 Servicio de Ginecología, Unidad de Patología Mamaria, Hospital Universitario de Caracas, MPPS, Caracas 1050, Venezuela

**Keywords:** breast cancer, human papillomavirus, prognostic factors, molecular subtypes

## Abstract

Breast cancer accounts for 16% of all female cancers worldwide, and in Venezuela, it is the leading cause of death among women. Recently, the presence of high-risk genotypes of human papillomavirus (HPV) has been demonstrated in breast cancer and has been associated with histopathological features of the tumours. In Venezuela, there is no study which determines the association between the presence of HPV in breast cancer and the histopathological features. The aim of this investigation is to evaluate the presence of HPV in the different types of breast cancer, according to their molecular classification, based on the expression of ER, PR, HER2 and Ki67. With this purpose in mind, we assessed the presence of the HPV genome in 24 breast cancer samples diagnosed with infiltrating ductal carcinoma, ductal carcinoma *in situ* (DCIS) and lobular carcinoma, by the INNO-LIPA genotyping extra kit and the evaluation of the markers ER, PR, HER2, and Ki67 by immunohistochemistry. The viral genome was found in 41.67% of the total number of samples, 51 being the most frequent genotype with 30.77%, followed by types 18 and 33, with 23.08%, respectively. Most tumours were found in the group of luminal A, with a low range of Ki67 expression. The presence of HPV in breast tumours could affect their growth pattern and metastatic power.

## Background

Breast cancer is the most common cancer in women in both developed and developing countries, representing 16% of all female cancers. It is estimated that over 508,000 women died in 2011 worldwide due to breast cancer. Although breast cancer is thought to be a disease of the developed world, almost 50% of breast cancer cases and 58% of deaths occur in less developed countries [1]. It is estimated that 45% of the 1.35 million new cases diagnosed each year, and more than 55% of breast cancer-related deaths occur in low- and middle-income countries. An estimated 1.7 million women will be diagnosed with breast cancer in 2020, a 26% increase from current levels, mostly in the developing world [2].

In Venezuela, according to the Yearbook Mortality of the Ministry of Popular Power for Health (MPPS) for 2011, breast cancer was the leading cause of cancer death among women, followed by cervical cancer, reporting 1.942 deaths, which represent 17.8% of all cancer deaths [3].

Breast cancer is classified according to the tumour node metastasis (TNM)-staging system based on tumour size (*T*), the presence or absence of metastases in the lymph nodes (*N*), and the presence of distant metastases (*M*) (American Joint Committee Cancer, AJCC, 2010), thus allowing for tumour stages [4].

Since late in the last century, molecular biology has provided a new perspective that has led to a different therapeutic approach, allowing a better understanding of the clinical prognosis and predicting response to systemic treatments [5].

The expression of hormone receptors, oestrogen receptors (ER), and progesterone receptors (PR), as well as overexpression or amplification of the human epidermal growth factor receptor 2 (HER2), have been identified as important predictors for patients with breast cancer. Currently, these markers are commonly used to define treatment and establish disease prognosis associated with clinical and pathological variables; such as lymph node involvement, tumour size, histological type, tumour grade, and surgical margins [6].

Approximately, two-thirds of breast tumours express ER and PR activation in the centre of the tumour, making them candidates for hormonal therapy. Another 20% have overexpression of HER2. These tumours may benefit from therapy with trastuzumab, a monoclonal antibody, that can be used alone or combined with chemotherapy [6, 7].

Breast tumours are classified into four subtypes, depending on their immunohistochemical profiles and based on deoxyribonucleic acid (DNA) microarrays. They are as follows: (a) Luminal A: ER+, PR+, HER2−, (b) Luminal B: ER+, PR+, HER2+, (c) Triple negative: ER−, PR−, HER2−, (d) HER2: ER−, PR−, HER2+ [8]. Luminal tumours have been associated with favourable prognosis with 80–85% survival at 5 years; while the triple negative and HER2 positive are associated with poor prognoses, with a high risk of recurrence in a period of 3 years and a high mortality rate in 5 years, with survival rate decreases to 50–60% [6, 8].

Despite advances in molecular techniques, the aetiological factor of breast cancer has not been determined. A risk factor that has been proposed for several years is the infection with human papillomavirus (HPV) in breast tissue, due to its high carcinogenic potential. This DNA virus expresses *E6* and *E7* oncogenes, which interact with p53 and pRB, respectively, facilitating the development of malignancies due to uncontrolled cell cycle activation and inhibition of apoptosis [9]. It is very common that the genome of the high-risk HPV types has been integrated into the host genome during carcinogenesis, which usually leads to a loss in the regulation of *E6* and *E7* transcription [10].

Reports on the distribution of the HPV infection in breast cancer are controversial. Several authors have found no relationship between the presence of HPV genome sequences and the development of breast cancer [11, 12, 13, 14, 15]. On the other hand, a moderate frequency of HPV infection in patients with breast cancer has been reported, ranging between 20–48%, while other authors reported a high frequency between 60–85% [16, 17, 18, 19, 20, 21, 22, 23, 24, 25, 26, 27, 28, 29].

This evidence allows a plausible relationship between breast cancer and the HPV infection, and it suggests that the viral infection may be a possible risk factor in the development of the disease that currently causes so many deaths worldwide among the female population. In Venezuela, there are no studies that correlate the presence of HPV with the prognostic markers of breast cancer, which is why this study aims to evaluate the presence of human papillomavirus in different types of breast cancer, according to the molecular classification based on the ER, PR, HER2, and Ki67 expression.

## Methods

### Study subjects

The total sample for the study included 24 patients diagnosed with ductal carcinoma *in situ* (DCIS), infiltrating ductal carcinoma and lobular carcinoma, in different stages according to the TNM classification. Fresh biopsies were obtained from patients who underwent surgery between February 2011 and March 2013, in the Gynecology Service, Breast Pathology Unit, of the University Hospital of Caracas, and cut into two sections. One section was frozen at −70°C for molecular analysis, and the other fragment was fixed in 10% neutral formalin and was paraffin-embedded. Each patient underwent a survey to collect clinical and socio-cultural data, and each was asked to sign an informed consent. This protocol was approved by the Bioethics Committee of the University Hospital of Caracas.

### DNA extraction and quality assessment of genetic material

To perform the DNA extraction from fresh breast biopsies, we used the QIAmp DNA Mini Kit (250) (QIAGEN. Hilden, Germany), following the manufacturer’s instructions. The quality of the isolated DNA was checked by polymerase chain reaction (PCR) of control genes with primers generating fragments of 100, 200, 300, 400, and 600 bp [30]. The reaction mixture was prepared using 0.4 μl of dNTPs (100 mM), 5 μl of each primer (100 pM), 6.5 μl of 10X buffer, 5 μl of MgCl_2_ (50 mM), 0.5 μl of Taq polymerase, and 27.6 μl of nuclease-free water, to a final volume of 50 μl. The amplification conditions were 7 min at 95°C, 35 cycles of 30 sec at 95°C, 40 sec at 60°C, and 40 sec at 70°C and a final amplification for 15 s at 70°C.

### HPV detection and genotyping

Detection and genotyping of the HPV genome from fresh breast biopsies was performed using the INNO-LIPA HPV Genotyping Extra test (Innogenetics, Belgium), following the manufacturer’s instructions. This test is a line probe assay, based on the reverse hybridization principle, designed for the identification of 28 different genotypes of the HPV by detection of specific sequences in the L1 region of the HPV. The assay uses the proven SPF10 primer set for the highly sensitive amplification of most clinically relevant HPV genotypes.

### Immunohistochemistry

The breast cancer biopsies were fixed in 10% buffered formalin and embedded in paraffin blocks. About 3 μm sections were obtained and placed on glass slides. The tissues were deparaffinized with xylene and rehydrated with serial dilutions of alcohol. Sections were incubated with primary antibody. The primary antibodies used were as follows: 1D5 to detect ER (1:50); and 1294 for PR (1:50) (Dako, Carpinteria, USA). The HercepTest (1:300) was used for the detection of HER2 and MIB-1 for Ki67 (1:100) (Dako, Carpinteria, USA). All antibodies were diluted in PBS. Then, a biotinylated anti-mouse secondary antibody (Dako Real EnVision, Denmark) was added, and the tissue sections were visualised by light microscopy. Appropriate controls were used.

Expression of Ki67 was evaluated according to the parameters published in Stathopoulos *et al* (2014) [31], where values ≤20% are considered low and values >20% are considered high.

### Statistical analysis

For statistical analysis, a Chi square test was performed to assess the independence of the variables, with the IBM SPSS Statistics software, version 2.0. Values less than or equal to 0.05 were considered statistically significant.

## Results

We evaluated the HPV presence in 24 samples of patients with breast cancer operated on at the University Hospital of Caracas, between the period of February 2011 and March 2013. The overall average age of patients with cancer was 56.75 ± 12.79 years (range: 37–84). The distribution of tumours according to the histological diagnosis was 83.33% for infiltrating ductal carcinomas and 8.33% for DCIS and lobular ductal carcinomas. The distribution, according to the tumour stages, were 54.16% for stage II, 25% for stage I, 12.5% for stage III and 8.33% for stage 0.

HPV detection was evaluated by molecular biology. We found 41.67% of the viral genome in breast tumours. The most frequent genotype was 51, followed by types 33 and 18, high oncogenic risk types, with a 30.77%, 23.08%, and 15.38%, respectively. The genotypes 6 and 11, low oncogenic risk types, were found in 15.38% each. 30% of the samples showed mixed infection. Most HPV-positive patients were within the age range of 51–60 years.

Table 1 presents the HPV frequency according to various clinicopathological features, including risk factors associated with the development of cervical cancer, which in turn, coincide with some associated factors with the development of breast cancer.

Breast tumours were classified based on the expression of the oestrogen and progesterone receptors, HER2 and the cell proliferation marker Ki67. Of the total sample, 54.17% was in the luminal A group, with an average value of 18% for Ki67 and 29. The luminal B group had 17%, with an average value of 15.71% for Ki67. The triple-negative group and HER2 were represented each by 8.33%, with an average value of 52.5% and 70% for Ki67, respectively.

According to this classification, the luminal A had 30.77% positivity for HPV, both low and high oncogenic risk in single and mixed infections. The luminal B group showed 57.14% positivity for HPV genotypes, equally low and high risk, but in single infections. In triple-negative patients, the presence of high oncogenic risk HPV was detected. The HER2 was the only group not associated with viral infection (Table 2). Although no statistically significant differences were found, the triple-negative group showed borderline significance.

## Discussion

In this study, the presence of HPV was detected in 41.67% of breast cancer fresh biopsies. This detection rate is within the published data worldwide (10–86%) [16, 17, 18, 19, 20, 24, 25, 28], where the works of Antonsson *et al* (2011) [32] and Glenn *et al* (2012) [33] show a frequency of 50% each. In Latin America, the rate is a little lower. It is reporting between 5–40% of positivity [26, 27, 28, 34], particularly in the case of Herrera *et al* (2013) [34], who report a 40% of positivity. Their samples were from metaplastic breast tumours, a subset of breast tumours, characterised for being more aggressive and worse prognosis.

Li *et al* (2011) [35] and Simões *et al* (2012) [36] conducted an epidemiological study based on the inclusion of case–control and cross-sectional studies, prospective and retrospective, where they evaluated the presence of HPV in clinical breast cancer, using PCR assays. The overall prevalence of HPV DNA in patients with breast cancer was 24.49% (range: 0–86.21%) and 23% (range: 13.4–42.9%), respectively. South America studies showed a significant lower prevalence of HPV, 15.1% and 16.67%, respectively. Finally, all case–control studies were pooled, and the OR was calculated, which showed that HPV-positive women are 6.31 [35] and 5.9 [36] times more likely to have breast cancer, compared with HPV-negative women.

HPV genotypes found in our samples were mainly high oncogenic risk types, with 51, 33 and 18 being the most frequent. Similar to the studies of Akil *et al* (2008) [22], Heng *et al* (2009) [25], Antonsson *et al* (2011) [32] and Glenn *et al* (2012) [33] report a higher frequency of these HPV genotypes, most of them carried out with samples of Australian patients and another previous study done with samples of Venezuelan patients [37]. In the rest of the papers published worldwide, it is reported that the HPV genotype 16 is the most common [23]. Based on these reports, it can be suggested that the distribution of HPV infection in patients with breast cancer appears similar to that observed in the cervix, which also depends on factors such as geographic, ethnic and racial differences, and pattern reported previously by Correnti *et al* (2011) [38].

Despite this support, some studies found no evidence of HPV in any of their breast tissue samples [11, 12, 13, 14, 15]. These differences may be explained by variations in the methodological approaches used to detect HPV. Moreover, PCR protocols showing a diverse sensitivity and specificity are currently used to determine HPV presence in tumoural tissue and a ‘gold standard’ protocol has not been defined [26]. Also, the use of a different primer set could contribute to this issue [23].

In this study, the HPV detection was performed using the INNO-LIPA HPV Genotyping Extra test. This permits ultrasensitive detection of a broad spectrum of HPV genotypes by PCR with biotinylated primers SPF10 that amplifies a 65 bp long region in the HPV L1 gene, and typing by a reverse hybridization (LiPA), identifying 28 high- and low-risk genotypes [24].

It is worth noting that several authors have suggested that the presence of HPV is associated with smaller breast tumours [24, 27, 32, 34], when compared with negative tumours for viral infection. In this work, despite the fact that we found no statistical significant relationship, HPV-positive breast tumours were found mainly among the groups T1 and T2 lesions with a maximum size of 50 mm. According to Antonsson *et al* (2011) [32], this feature might imply that the presence of HPV could have an important role in the growth pattern and metastatic potential of breast carcinomas associated with a better prognosis, as in the case of tumours of the head and neck.

Regarding the molecular classification of breast cancer, samples from this study mainly corresponded to luminal group A, which have a high expression of oestrogen receptors and low levels of proliferation, resulting in low mitotic tumour cells and excellent prognosis [39], followed by luminal B tumours, triple negative, and HER2. This is consistent with those reported by Uribe *et al* (2010) [40], who reported a 60.63% rate of luminal A tumours in patients of Estado Lara, Venezuela. Stathopoulos *et al* (2014) [31] recommend using Ki67 as a predictive factor in luminal A type tumours, reporting values ≤20% in 99%. For luminal B, Ki67 distributed values found between low and high values, whereas in triple negative and HER2, Ki67 values were above 20%. The samples evaluated in our study had a similar behaviour, being able to establish a relationship between the molecular subtype and cell proliferation marker.

There are few studies that have evaluated the relationship between the presence of HPV and prognostic factors of breast cancer. Henning *et al* (1999) [17] and Damin *et al* (2004) [19] found no statistically significant differences between these variables. However, Kroupis *et al* (2006) [41] reported that HPV-positive tumours have lower positivity for RE, higher Ki67 expression and high histological grade. These authors indicated the need for further studies to evaluate the possibility that the proliferative potential and aggressive tumour phenotype is directly due to the presence of high-risk HPV at the time of carcinogenesis in the breast.

Currently, the mechanism of HPV infection in the breast is uncertain. Due to the fact that the HPV life cycle occurs in epithelial layers, bloodstream viraemia is not a possible event [34]. The following two hypotheses are considered: (1) infection via the nipple skin, as demonstrated in the work of DeVilliers *et al* (2005) [20], which indicate the presence of the HPV genome in the tumour tissue, as in the nipple, proposing a retrograde ductular pattern of viral spread. (2) Haematologic infection being shown by the presence of mononuclear cells carrying HPV, isolated from women with cervical cancer [42], HIV-infected children [43] and healthy donors [44]. Additionally, other authors reported the presence of HPV-16 in breast tumours from patients with a history of cervical lesions caused by this viral type [17, 45], which suggests that malignant transformation is the result of a cell transfection by the plasma flow from the primary tumour.

More recently, several studies have converged upon the innate immune DNA cytosine deaminase APOBEC3B (A3B) as a significant source of genomic uracil lesions and mutagenesis in multiple human cancers, including those of the breast, head, and neck, cervix, bladder, lung, ovary, and other tissues. This enzyme belongs to a protein family that has broad and overlapping functions in innate immunity by restricting viruses, transposons and other foreign DNA elements [46].

Vieira *et al* (2014) [46] showed that the upregulation of A3B mRNA is directly induced by the high-risk E6 HPV protein, transfecting the full-length HPV genome or a full-length HPV genome containing a stop codon within the E6 open reading frame in cell lines. Then, it was probed in samples of patients with head and neck cancer, where reported overexpression of A3B in HPV-positive tumours.

Ohba *et al* (2014) [47] conducted a similar work, but in the cell line MCF10A, to determine whether HPV can be the starting point of carcinogenesis in breast cancer, by overexpression of A3B, allowing excessive mutations, dysregulated cell cycle and subsequent transformation. Their results show that HPV infection induces overexpression of A3B and infected cells represent more malignant phenotype than parental cells. These malignant phenotypes were largely abrogated when A3B was knocked down in HPV-infected cells.

## Conclusions

The HPV genome was found in 41.67% of all breast cancer samples, mainly identifying high-risk oncogenic genotypes. Most of the breast tumours were luminal A. There was no statistically significant difference between the presence of HPV and the prognostic factors. However, further studies involving a larger number of samples and the evaluation of other parameters are needed, such as viral load, integration of the viral oncogenes and several pathways for the introduction of genomic instability.

## Conflicts of interest

The authors declare that they have no conflicts of interest.

## Figures and Tables

**Table 1.** Clinical and histopathological features of breast tumours, according to the presence of HPV.

	HPV- (*n* = 14) *n* (%)	HPV+ (*n* = 10) *n* (%)	p^*^
Age	56 (37–84)	57.8 (40–73)	**0.735**
Number of couples	1.94 (0–4)	1.6 (0–3)	
Number of births	2.57 (0–9)	2.5 (0–6)	**0.665**
History of cancer	9 (64.29)	5 (50)	**0.484**
Alcohol	8 (57.14)	5 (50)	**0.729**
Tobacco	7 (50)	7 (70)	**0.327**
Oral contraceptives	5 (35.71)	4 (40)	**0.831**
Histology			
DCIS	2 (14.29)	0	**0.454**
IDC	11 (78.57)	9 (90)	
LC	1 (7.14)	1 (10)	
TNM stage			
E0	2 (14.29)	0	**0.209**
EI	3 (21.43)	3 (30)	
EII	6 (42.86)	7 (70)	
EIII	3 (21.43)	0	
Tumour size			
Tis	2 (14.29)	0	**0.363**
T1	3 (21.43)	4 (40)	
T2	6 (42.86)	4 (40)	
T3	1 (7.14)	2 (20)	
T4	2 (14.29)	0	

HPV: human papillomavirus;

DCIS: ductal carcinoma *in situ*;

CDI: infiltrating ductal carcinoma;

CL: lobular carcinoma.

* *p* < 0.05 (No statistically significant differences were found).

**Table 2.** Molecular subtypes of breast tumours, according to the presence of HPV.

	Luminal A (*n* = 13) *n* (%)	Luminal B (*n* = 7) *n* (%)	Triple- (*n* = 2) *n* (%)	HER-2 (*n* = 2) *n* (%)
HPV+	4 (30.77)	4 (54.14)	2 (100)	0
HPV-	9 (69.23)	3 (42.86)	0	2 (100)
p^*^	**0.239**	**0.324**	**0.081**	**0.212**

HPV: human papilloma virus.

* *p* < 0.05.
